# Evidence of rift valley fever seroprevalence in the Sahrawi semi-nomadic pastoralist system, Western Sahara

**DOI:** 10.1186/1746-6148-10-92

**Published:** 2014-04-24

**Authors:** Antonello Di Nardo, Davide Rossi, Saleh M Lamin Saleh, Saleh M Lejlifa, Sidumu J Hamdi, Annapia Di Gennaro, Giovanni Savini, Michael V Thrusfield

**Affiliations:** 1 Institute of Biodiversity, Animal Health and Comparative Medicine, College of Medical, Veterinary and Life Sciences, Glasgow University, Glasgow, UK; 2 The Pirbright Institute, Pirbright, Woking, Surrey, UK; 3 Africa 70 (International Non-Governmental Organization), Monza, MI, Italy; 4 Sahrawi Veterinary Services, Ministry of Public Health, Sahrawi Arab Democratic Republic, Rabouni, Algeria; 5 Istituto Zooprofilattico Sperimentale dell’ Abruzzo e del Molise “G. Caporale”, Teramo, Italy; 6 Veterinary Clinical Sciences, Royal (Dick) School of Veterinary Studies, College of Medicine and Veterinary Medicine, The University of Edinburgh, Easter Bush Veterinary Centre, Roslin, Midlothian, UK

**Keywords:** Rift Valley fever, Small ruminants, Camels, Semi-nomadic pastoralist system, Epidemiology, Sahrawi, Western Sahara

## Abstract

**Background:**

The increasing global importance of Rift Valley fever (RVF) is clearly demonstrated by its geographical expansion. The presence of a wide range of host and vector species, and the epidemiological characteristics of RVF, have led to concerns that epidemics will continue to occur in previously unaffected regions of Africa. The proximity of the Sahrawi territories of Western Sahara to endemic countries, such as Mauritania, Senegal, and Mali with periodic isolation of virus and serological evidence of RVF, and the intensive livestock trade in the region results in a serious risk of RVF spread in the Sahrawi territories, and potentially from there to the Maghreb and beyond. A sero-epidemiological survey was conducted in the Saharawi territories between March and April 2008 to investigate the possible presence of the RVF virus (RVFV) and associated risk factors. A two-stage cluster sampling design was used, incorporating 23 sampling sites.

**Results:**

A total of 982 serum samples was collected from 461 sheep, 463 goats and 58 camels. Eleven samples (0.97%) tested positive for IgG against the RVFV. There were clusters of high seroprevalence located mostly in the Tifariti (7.69%) and Mehaires (7.14%) regions, with the Tifariti event having been found in one single flock (4/26 positive animals). Goats and older animals were at a significantly increased risk being seropositive (p = 0.007 and p = 0.007, respectively).

**Conclusion:**

The results suggest potential RVF activity in the study area, where intense livestock movement and trade with neighbouring countries might be considered as a primary determinant in the spread of the disease. The importance of a continuous field investigation is reinforced, in light of the risk of RVF expansion to historically unaffected regions of Africa.

## Background

Rift Valley fever (RVF) is an acute arthropod-borne disease affecting a wide range of animals, ranging from rodents to camels [1]. However, the most economically-important hosts are sheep, goats and cattle, in which high neonatal mortality and abortion in pregnant animals occur [2]. The Rift Valley fever virus (RVFV) (family *Bunyaviridae*, genus *Phlebovirus*) is also one of a handful of viruses that cause a mild to moderate influenza-like syndrome in humans, with severe complications, such as lesions of the macula and the retina, meningo-encephalitis and haemorrhagic disease, occurring in some of the patients [3].

Rift Valley fever can induce substantial economic losses through high abortion rates and death of neonates and young animals during epidemics. It also incurs heavy control costs, including those of culling and compensation, vaccination and vector control [4]. Additionally, its presence in a country that exports livestock can cause trade bans with severe financial and welfare effects for pastoral communities that are dependent on livestock for their livelihoods [5].

Several African sub-Saharan tropical and sub-tropical countries have reported outbreaks of RVF, and the disease is encountered in endemic and epidemic forms along the east and south coast of Africa, in West Africa, and in Madagascar [6]. The virus has spread as far North as Egypt and more recently an outbreak occurred in the Arabian Peninsula [7,8]. The ability of RVF to move outside traditionally endemic countries, even out of the African continent, lies in the fact that a large range of arthropod vectors is capable of transmitting the virus; moreover, the level of viraemia in ruminants is sufficiently high to infect mosquitoes [9]. Given the right conditions, this transboundary animal disease has the potential to spread over large distances, deserving further consideration in a world where globalisation of trade and short transportation times are now common.

The increasing global importance of RVF is clearly demonstrated by its geographical expansion. The presence of a wide range of host and vector species, and the epidemiological characteristics of RVF, has led to concerns that epidemics may occur in previously unaffected regions of Africa, and beyond [10]. Recently, Clements and others [6] presented the first atlas of RVF seroprevalence in Africa, reviewing data from surveys conducted in several African countries. However, no information was available from many areas: these included all of North Africa but Egypt, Mali, much of the Western Africa south of the Sahelian zone, the Democratic Republic of Congo, much of Eastern Africa, the Horn of Africa and parts of Southern Africa [6].

The current situation in the Sahrawi Arab Democratic Republic (SADR) held territory and the whole Western Sahara (WS) region reflects this paucity of information. Indeed, the SADR can be considered as an ‘epidemiological question mark’ in West Africa. The animal health institutions, and therefore disease surveillance, are still embryonic. In addition, the prevalence and distribution of a number of transboundary animal diseases of major international concern, such as bluetongue, foot-and-mouth disease, and RVF are not known. The proximity of the SADR to endemic countries, such as Mauritania, Senegal, and Mali with periodic isolation of virus and serological evidence of RVF, and the intensive livestock trade between the SADR and these countries poses a serious risk of RVF spread in the SADR, and potentially from there to the Maghreb and beyond. A sero-epidemiological survey was thus conducted in the SADR between March and April 2008 to investigate the possible presence of the virus and its associated risk factors.

## Methods

### Study area

**Figure 1:**
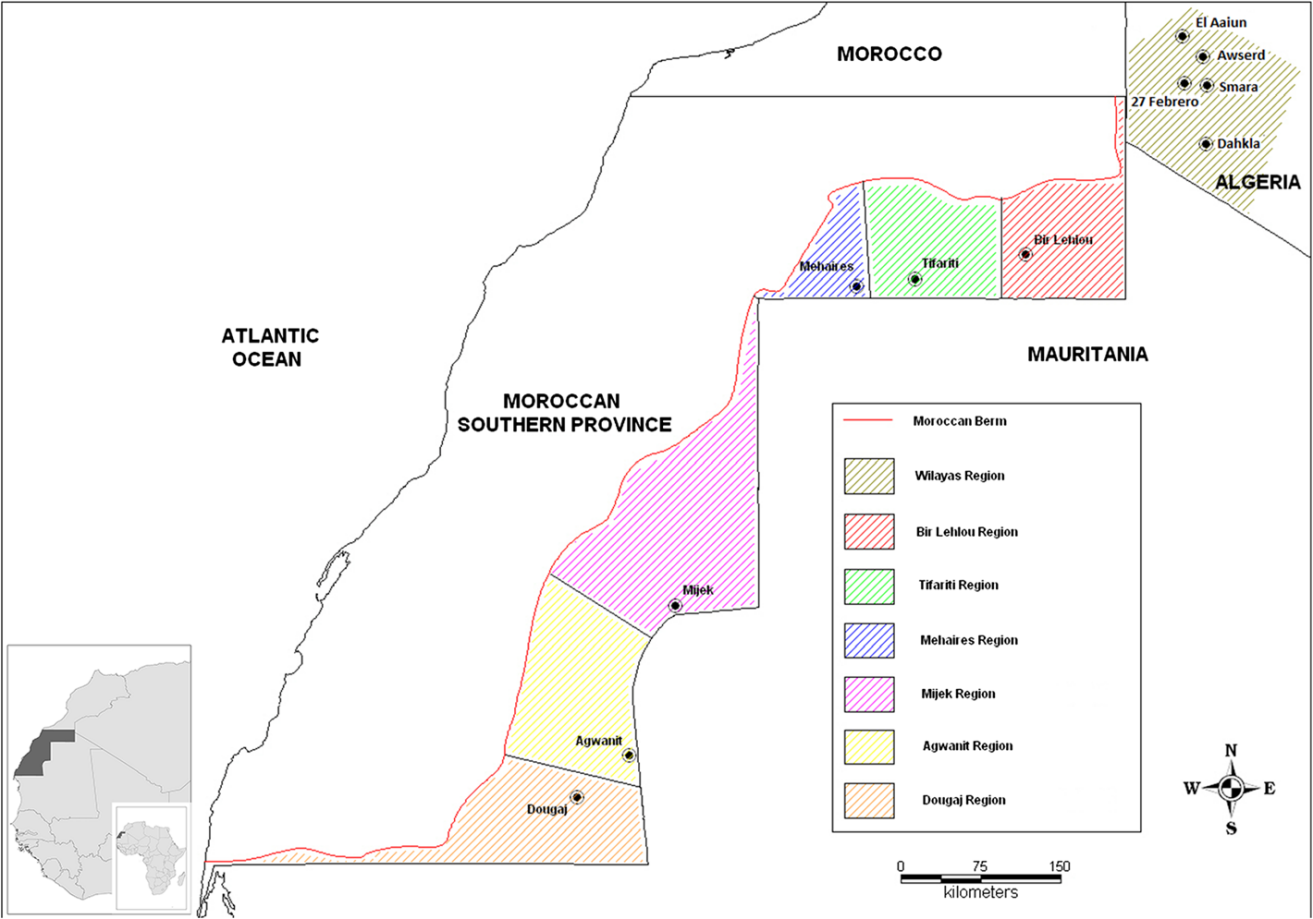
Geographical allocation of the study area.

The WS is located at 8°40'-17°06'W, 20°46'-27°40'N. Sahrawi, literally ‘people from the desert’, is the name given to tribes of nomadic and pastoral people who traditionally inhabited WS [11]. In 1975, as a consequence of the military occupation of Western Sahara by Mauritanian and Moroccan military forces, about 70,000 Sahrawi fled into Algeria [12,13] where they gathered in refugee camps in the Tindouf province. Subsequently, a long political process led to the establishment of a formally proclaimed government-in-exile, the SADR. The SADR has political control of the eastern part of WS (also referred as ‘liberated territories’ or ‘free zone’), divided into six military regions (Bir Lehlou, Tifariti, Mehaires, Agwanit, Dougaj), and the refugee camps (or “wilayas”, El Aaiun, Awserd, Smara, Dahkla and 27 Febrero), located in the desert plateau of Hamada within the Tindouf province of western Algeria (Figure 1). The total livestock population in the wilaya region is about 63,000, whereas in the ‘liberated territories’ the number is estimated to be approximately 140,000 [14]. Livestock comprise sheep, goats and camels, reared under semi-nomadic conditions by Sahrawi pastoralists between the wilaya and the ‘liberated territories’ regions. The SADR economy relies mostly on the livestock trade with neighbouring countries, selling animals in livestock markets present in the refugee camps or by import/export of animals by trading routes, largely with Mauritania, Mali, and Algeria. In this context, livestock trade represents one of the most lucrative activities for Sahrawi, as well as one of the factors that increase cash flow in the camps [15].

### Study design

The sample size was calculated from the 2007 census animal population data [14]. However, these data considered only the total number of animals present in each wilaya and military region and so it was not possible to set up a list of all herds present in the territory.

Two-stage cluster sampling therefore was used [16]. A cluster was defined as a settlement, watering point or grazing area where animals were expected to be found. Due to the lack of an exhaustive list of these locations in the SADR, high mobility of pastoral herds, unpredictability of their movements and temporary character of nomadic settlements, classic random selection of clusters was not feasible. Therefore, the sample size was calculated considering a fixed number of clusters. The rationale behind the cluster definition was guided by the RVF epidemiology (i.e. vector biology and virus ecology), by local ecosystem distinctions, as well as by the peculiarities of the Sahrawi pastoralist system. Hence, the clusters were selected according to animal population and ecosystem distinctions between and within the wilayas and the ‘liberated territories’ regions. Thus the primary sampling unit represented the livestock system and the ecosystem present in each cluster. Eleven target clusters were identified: one cluster for each wilaya or refugee camp (n = 5) and one cluster for each military region (n = 6) (Figure 1). A fixed radius was defined for each target cluster in order to determine the geographical area within which the sampling was conducted. Because the radius depended on the density and mobility of the pastoral herds, a 5 km radius was defined for the wilayas clusters, whereas a 20 km radius was defined for the military regions.

### Sample size and sampling assumptions

Where RVF is present in its endemic form, a range of 10-35% seroprevalence has been most frequently reported in outbreaks. As RVFV IgG antibodies are proven to be lifelong in clinically infested animals [17], similar levels of prevalence were expected to be in the SADR at the time of the survey. Therefore, it was assumed that the seroprevalence was low. For the sample size calculation, a two-stage cluster sampling formula for a 95% confidence interval was used [16] as $n=\frac{1.96^{2}gP_{\text{exp}}\left(1-P_{\text{exp}}\right)}{gd^{2}-1.96^{2}V_{c}}$, where *n* = required sample size, *g* = number of clusters to be sampled, *P*_exp_ = expected prevalence, *d* = absolute precision, *V*_*c*_ = between-cluster variance. The sample size was calculated assuming a *P*_exp_ = 15%, with *d* = ±5%, and *Vc* = 0.0039 (estimated from a previous RVF survey carried out in Somalia in 2003) (Tempia S., personal communication). The target population consisted of resident sheep, goats and camels. These sampling assumptions were used to calculate the sample sizes for each of the species. In each of the 11 clusters, a mean of 41 individuals of each species, i.e. sheep, goats and camels, respectively, were to be randomly sampled totalling 1334 serum samples (Table 1). In addition, a further 5% per cluster was sampled to cater for poor blood clotting.

**Table 1:** **Proportional allocation of calculated sample size by animal species (95% CI, 5% *d*, 15% *P***_**exp**_**, 0.0039 *V***_**c**_**)**

**Species**	**Total population (2007 census data)**	**Sample size required**	**Sample size per 11 cluster**
Sheep	62681	443.1	40.3
Goats	51649	443.8	40.3
Camels	26175	447.5	40.7
**TOT**	140505	1334.4	121.3

The single sample size obtained for each specie was tested in CSurvey 2.0 [18] to assess the acceptability of the sample sizes calculated. All the parameters entered in the program were in agreement with the sample size computed.

Animals were randomly selected from herds located within each cluster without replacement, using tables of random numbers generated according to the total population data via Survey Toolbox 1.0b [19].

### Screening for RVF antibodies in the SADR and collection of epidemiological and RVF risk-related data

The sero-epidemiological survey was implemented during March-April 2008. All the blood samples were refrigerated at 4°C during collection and centrifuged in the field at the military bases of the ‘liberated territories’. Then, the sera obtained were immediately frozen at −21°C. An adequate cold chain facilitated the retrieval of all sera for subsequent analysis. Additional epidemiological data, such as abnormal abortion rates, mortality in newborn animals, as well as information on favourable ecological conditions for vector proliferation and disease spread, were recorded along with blood samples through semi-structured interviews [20] with Sahrawi herders. A target group of pastoralists was identified and semi-structured interviews were carried out after blood sampling. In every case, prior informed consent was obtained before the interview and the sampling procedures were undertaken, and participants were given an explanation of the methodology, aims, and expected outcomes of the study, following the ethical guidelines adopted by the American Anthropological Association [21]. This study was performed in adherence with the Animal Research: Reporting of *In Vivo* Experiments (ARRIVE) guidelines [22], where high standard (best practice) of veterinary care was adopted during the field interventions.

### Testing methodology and data analysis

The serum samples were screened for RVF antibodies using the inhibition-ELISA test (Biological Diagnostic Supplies Ltd., UK) as a kit [23]. The tests were carried out in June 2008 at the Virology Department laboratories of the Istituto Zooprofilattico Sperimentale dell’Abruzzo e del Molise, Italy, according to the manufacturer’s protocol. Plates were read by measuring Optical Density (OD) at a wavelength of 405 nm, and then expressed as a Percentage Inhibition (PI) as $\mathrm{PI}=100-\frac{\mu_{\mathrm{ts}}}{\mu_{\mathrm{nc}}}100$, where *μ*_*ts*_ = mean net-OD of test sample, *μ*_*nc*_ = mean net-OD of negative control, to define the positive/negative sera, following the cut-off values set for each animal species provided by the protocol [23]. PI values ≥38.4, ≥41.4 and ≥36.1 were considered positive for sheep, goats and camels, respectively. More specifically, a PI value of ≥40 but ≤75 was considered a weak positive result and a PI value of ≥75 was considered a strong positive result. The inhibition-ELISA has sensitivity and specificity parameter values ranging from 99.56% to 100% and 99.29% to 100%, respectively [23]. Accordingly, if the test is used in the SADR the probability (*P*) that a positive animal (*D*^+^) escapes detection would vary between 0.009 and 0 (calculated as the complementary probability of the predictive value of a negative test (*NPV*), assuming the expected prevalence of 15%, *P*(*D*^+^) = 1 − *NPV*) [16]. ELISA results were stored in an Excel 2010 (Microsoft Corporation) spreadsheet along with species, age, sex, sampling location, and Global Positioning System (GPS) data. The data were stratified by region (wilayas were considered as a single region) and then by sampling site. To account for differential probabilities of selection due to the study design and to ensure proper survey estimate, the sampling base weight was calculated for each sampling site as the inverse of the first stage selection probability assigned to a sampled cluster as $BW_{c}^{i}=\frac{M}{n\times m_{i}}$, where *n* is the number of sampled clusters, *m*_*i*_ the measure of the size for the *i*th cluster and $M=\sum_{i=1}^{N}m_{i}$[24]. Data analysis was performed using Stata 12.1 SE (StataCorp. LP), where confidence intervals were calculated using the Agresti Coull test for binomial distributed variables [25]. Univariable analysis was performed by the Adjusted-Wald test, considering the effect of species, age, and location (region and sampling site levels) on the RVF seroprevalence observed. The sampling base weight was taken into account for the analysis of the data. Sensitivity and specificity of the test [23] were used to calculate the true prevalence from the observed test prevalence [26]. Inter-cluster variability was estimated by calculating the Design Effect (DEFF) and the Rate of Homogeneity (ROH) [27]. The maps were constructed using ArcGIS 10.1 (Environmental Systems Research Institute, Inc.).

## Results

### Rift valley fever seroprevalence

**Figure 2:**
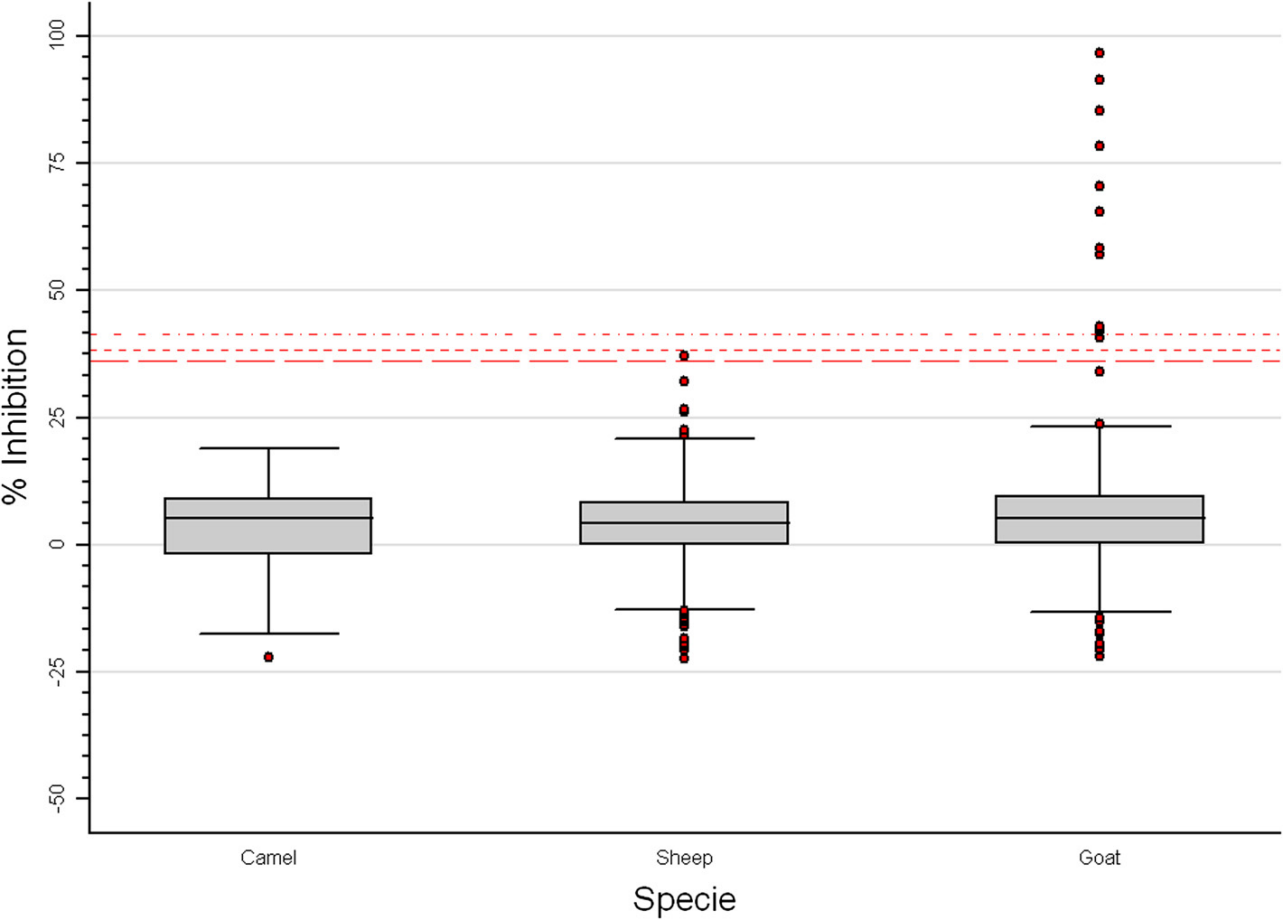
**Box plot of inhibition-Elisa test results for all species sampled.** Percentage of Inhibition values of ≥36.1 for camel (long-dash red line), ≥38.4 for sheep (short-dash red line) and ≥41.4 for goat (dash-dot red line) indicate a positive result.

**Figure 3:**
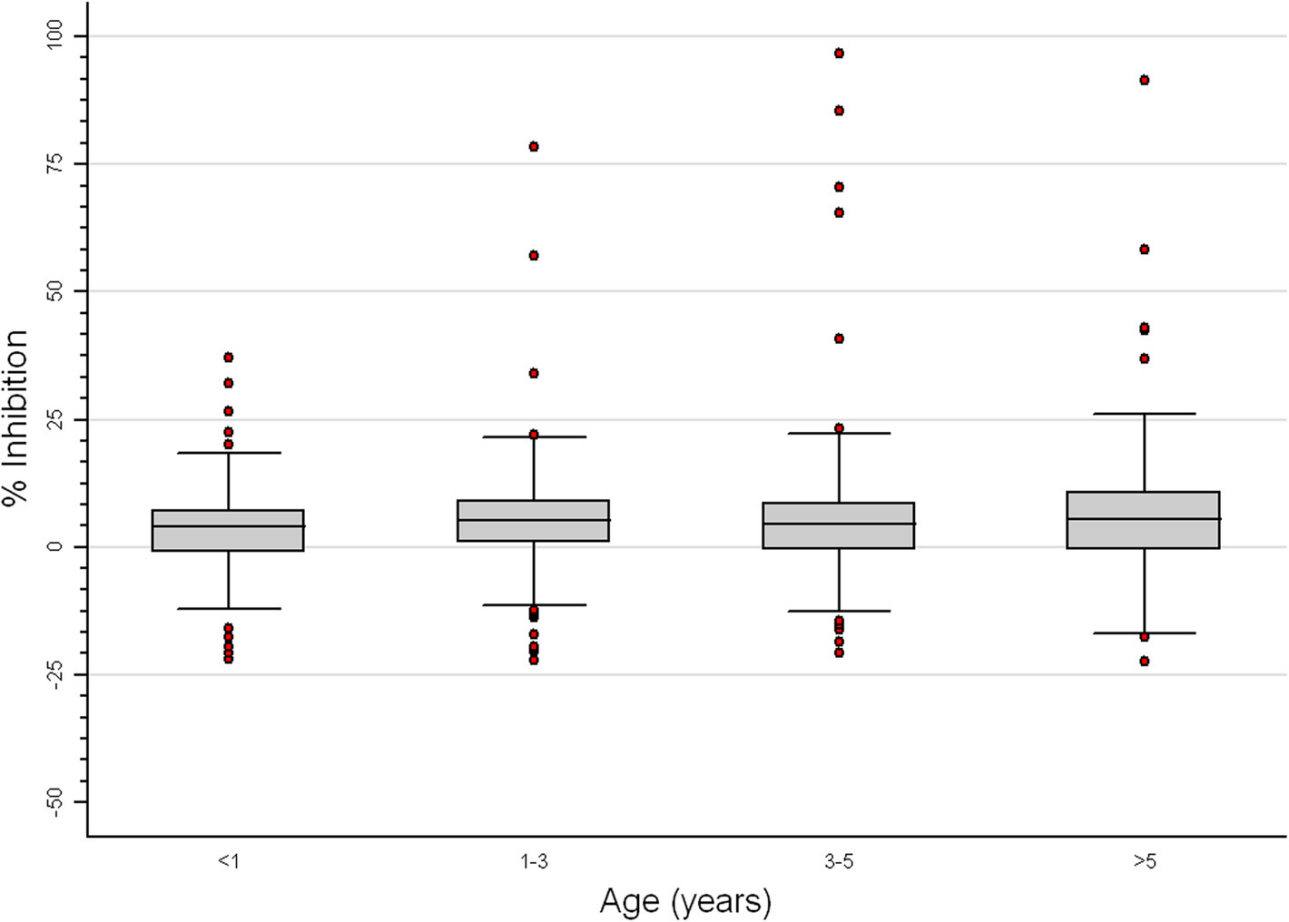
Box plot of inhibition-Elisa test results versus age groups.

Field constraints limited the scope of the survey and only a total of nine hundred and eighty two animals were sampled in 23 sites (representing 74% of the expected sample size). Only 58 camels were sampled (representing 13% of the expected sample size). The relatively low figure for sampled camels was due to different factors, such as religious beliefs and sometimes scepticism of camel owners in relation to blood extraction, as well as objective limitations in locating camels’ owners and obtaining permission and help (given the extensive pastoral system present in some areas and the reluctant behaviour of many camels to immobilization). All sites sampled were within a 20 km of radius of all target clusters. Eleven out of 982 samples tested positive for IgG against RVFV (0.97%; 95% CI 0.33 – 2.85). During the field survey no RVF clinical signs were observed. The seroprevalence at regional level, as well as the range of within-site prevalence, are summarised in Table 2. A higher than the observed overall prevalence value was reported in the Tifariti region (4.96%, 95% CI 1.88 – 12.44), with a total of four positive animals. In the other regions, a uniform pattern of prevalences was revealed, with some isolated cases in the wilayas (0.92%, 95% CI 0.25 – 3.38), Bir Lehlou (0.69%, 95% CI 0.09 – 4.83), Mehaires (1.22%, 95% CI 0.17 – 8.21), and Dahkla (1.75%, 95% CI 0.24 – 11.48) regions. Effects of species, age and region variables were found to be of statistical significance on seroprevalence estimates (p = 0.007, p = 0.007 and p = 0.0001, respectively) (Figures 2 and 3), where higher prevalence values were reported in goats and animals older than 3ys. No significant difference between sexes was observed (p = 0.23).

**Table 2:** Observed and true Rift Valley fever animal prevalence, associated exact 95% Confidence interval for two-stage cluster sampling and within sampling site prevalence by region

**Region**	**Positive animals/No sampled**	Observed [true] prevalence* (%)	**95% Confidence interval (%)**	**Within site prevalence range (%)**
Wilayas	4/356	0.92 [0.95]	0.25 – 3.38	0 – 2.47
Bir Lehlou	1/90	0.69 [0.70]	0.09 – 4.83	0 – 1.14
Tifariti	4/89	4.96 [5.29]	1.88 – 12.44	0 – 7.69
Mehaires	1/84	1.22 [1.27]	0.17 – 8.21	0 – 7.14
Mijek	0/81	0 [0]	0 – 0	0 – 0
Agwanit	0/94	0 [0]	0 – 0	0 – 0
Dougaj	0/85	0 [0]	0 – 0	0 – 0
Dahkla	1/103	1.75 [1.84]	0.24 – 11.48	0 – 1.75
**TOT**	11/982	0.97 [1.00]	0.33 – 2.85	0 – 7.69

*Adjusted Wald test between region (d.f. 7, 974) (p = 0.0001).

A DEFF of 4.7 indicated strong clustering, whereas the ROH value of 0.088 revealed low homogeneity within clusters. Moreover, the DEFF and the ROH values were in agreement with those reported by McDermott and Schukken [28], and by Otte and Gumm [29], where the ROH values lay between 0.05 and 0.10 and the upper estimate of ROH did not exceed 0.20. The sample of 23 clusters was smaller than the minimum of 30 recommended by Cochran [30] to rely upon the cluster means having a Normal distribution. However, on inspection of the former, the distribution was smooth and symmetric; thus, the confidence intervals could be considered to be valid, albeit approximate [16].

### Spatial distribution

**Figure 4:**
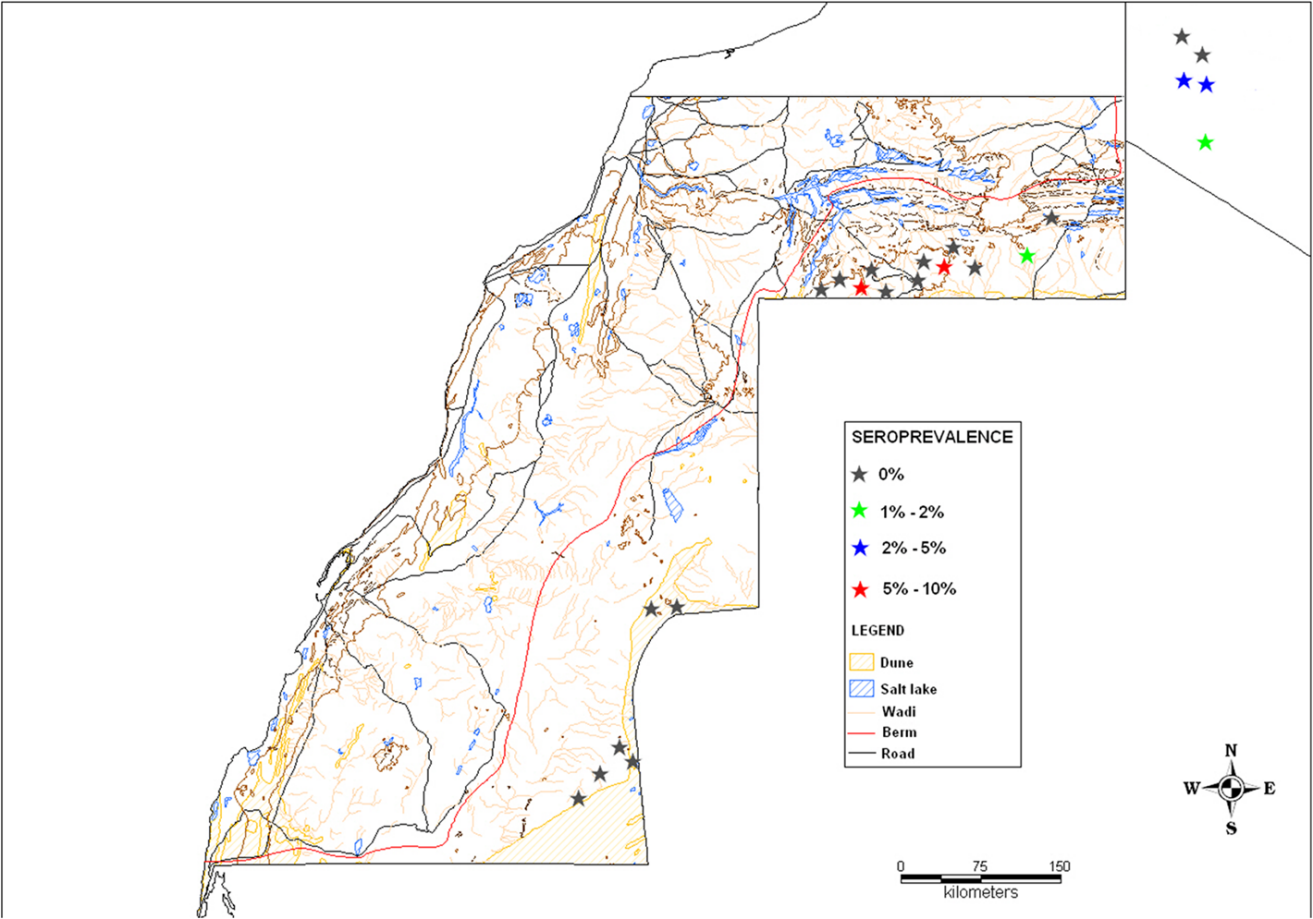
Observed Rift Valley fever sero-prevalence distribution in the study area.

Although the results of the RVF survey in SADR showed a low or zero prevalence in most regions, a relatively higher prevalence was concentrated in the Tifariti and Mehaires regions. Indeed, the breakdown of serological results by sampling sites (Table 3) revealed a higher prevalence at the Tifariti sampling site 10 (7.69%, 95% CI 2.91 – 18.80) and at the Mehaires sampling site 15 (7.14%, 95% CI 1.00 – 36.95) (Figure 4). The presence of RVFV antibodies reported in Tifariti and Mehaires regions provides evidence of previous exposure of goats to the RVFV: seroprevalence in goats’ flocks were reported reaching 15.38% and 14.29%, respectively. In addition, the seropositive cases in Tifariti originated from a single flock (4/26 positive animals), where high IgG levels were observed in two of these animals (PI values >91). Within the wilayas region, seropositive cases were reported in 27 Febrero (2.47%, 95% CI 0.62 – 9.38) and Smara (2.35%, 95% CI 0.59 – 8.95); two positive goats from 27 Febrero belonged to the same flock, in which the seroprevalence reached 4.88% with high IgG antibody titres (PI values of 78.47 and 85.32, respectively). Differences in seroprevalence estimates between sampling sites were found to be statistically significant (p = 0.0001).

**Table 3:** Observed and true Rift Valley fever animal prevalence, associated exact 95% Confidence interval for two-stage cluster sampling and within specie prevalence by sampling site

**Site**	**Location**	**Positive animals/ No sampled**	Observed [true] prevalence* (%)	**95% Confidence interval (%)**	**Within-species prevalence range (%)**
1	27 Febrero	2/87	2.47 [2.61]	0.62 – 9.38	0 – 4.88
2	Smara	2/92	2.35 [2.49]	0.59 – 8.95	
3	Awserd	0/86	0 [0]	0 – 0	0 – 0
4	El Aaiun	0/91	0 [0]	0 – 0	0 – 0
5	Bir Lehlou	1/60	1.14 [1.19]	0.16 – 7.79	0 – 2.27
6	Bir Lehlou	0/6	0 [0]	0 – 0	0 – 0
7	Bir Lehlou	0/10	0 [0]	0 – 0	0 – 0
8	Bir Lehlou	0/14	0 [0]	0 – 0	0 – 0
9	Tifariti	0/23	0 [0]	0 – 0	0 – 0
10	Tifariti	4/61	7.69 [8.22]	2.91 – 18.80	0 – 15.38
11	Tifariti	0/5	0 [0]	0 – 0	0 – 0
12	Mehaires	0/17	0 [0]	0 – 0	0 – 0
13	Mehaires	0/21	0 [0]	0 – 0	0 – 0
14	Mehaires	0/15	0 [0]	0 – 0	0 – 0
15	Mehaires	1/20	7.14 [7.63]	1.00 – 36.95	0 – 14.29
16	Mehaires	0/11	0 [0]	0 – 0	0 – 0
17	Mijek	0/15	0 [0]	0 – 0	0 – 0
18	Mijek	0/66	0 [0]	0 – 0	0 – 0
19	Agwanit	0/38	0 [0]	0 – 0	0 – 0
20	Agwanit	0/56	0 [0]	0 – 0	0 – 0
21	Dougaj	0/42	0 [0]	0 – 0	0 – 0
22	Dougaj	0/43	0 [0]	0 – 0	0 – 0
23	Dahkla	1/103	1.75 [1.84]	0.24 – 11.48	0 – 5.26
**TOT**		11/982	0.97 [1.00]	0.33 – 2.85	0 – 15.38

*Adjusted Wald test between sampling sites (d.f. 22, 974) (p = 0.0001).

## Discussion

The cluster sampling method applied has resulted in effective and reliable screening in the WS context, confirming the utility of the survey structure: the sample area defined for each cluster enabled sampling of the SADR nomadic and semi-nomadic systems, where flocks are typically scattered throughout the territory.

Although the overall RVF prevalence (0.97%) is not alarming, the presence of a cluster with high prevalence levels (Tifariti site 10, 7.69%; Mehaires site 15, 7.14%) deserves more attention because it may suggest local RVF activity.

It is not possible to conclude that RVFV is endemic in the region, although the presence of IgG antibodies is unquestionable. The high antibody levels found in Tifariti suggests previous exposure of the sampled flocks to the virus. As reported by Sahrawi during interviews, mosquitoes seem to be present throughout the year with some population increase mainly after rainfall. In fact, the survey was carried out two years after the rainfall event in 2006 and, if the climatic data for the corresponding period were analysed, also considering the Normalized Differentiation Vegetation Index [31], a correlation would likely be established with persistent flooding of mosquito habitats favourable to emergence of infected vectors in this area. High rainfalls are reported by Sahrawi at intervals of approximately 4 years (1986, 1990, 1994, 1998, 2000, 2003, 2006) sometimes resulting in flooding as, for example, in 2006. Indeed, rains are not evenly distributed across the territory, and it is worth noting that the 2006 rains fell mostly on the northern part of the ‘liberated territories’ and on the refugee camps. Large areas were temporarily flooded, therefore possibly creating conditions for vector propagation and RVF diffusion. These areas correspond to those sampling sites where presence of antibodies against the RVFV has been detected. Also during autumn 2008, rains occurred copiously, suggesting that rain intervals are shortening. Moreover, livestock are often concentrated where watering points and grazing areas are found, adding to the epidemiological conditions favourable for viral circulation. RVF seroprevalence was found to be significantly higher in older animals (p = 0.007), supporting the hypothesis of previous exposure to the RVFV. High antibody levels were reported in goats (p = 0.007) at Tifariti site 10 (15.38%) and Mehaires site 15 (14.29%). Considering that this species is more susceptible to RVFV [1], it probably constituted an indicator of low-level viral circulation in the Tifariti region. In addition, the herdsman of the goat flock at Tifariti site 10 reported past abortion events, and in particular, some cases after the rainfall in 2006: this constitutes useful information to be linked to the occurrence of RVF.

Therefore, the rainfall event in 2006 may have been the trigger for low-level RVFV circulation, but with no observable epidemic. Indeed, the WS ecosystem could constitute a suitable environment for virus maintenance and low-level circulation, but only in particular conditions could this lead to an epidemic, for example, if there is unrelenting rainfall or multiple rainfalls within a short period, leading to massive mosquito breeding events and then amplification of viral transmission. However, climatic variability due to *El Niño* events could predispose to that situation [32]. As to the introduction of RVFV in the region, some speculative hypotheses are: i) introduction through viraemic animal trading from neighbouring endemic countries or ii) wind spread of infected vectors from Mauritania, as reported in previous studies [1]. However, the first hypothesis is more likely.

The presence of IgG antibodies against the RVFV in four animals within the wilayas territories (2 in 27 Febrero, 2.47%; 2 in Smara, 2.35%) is evidence of the introduction of RVFV in the SADR. Noteworthy, the wilayas are settled and located in the Algerian territory considering, therefore, the first report of RVF seroprevalence in the Maghreb region. There is high meat consumption in the camps, and in order to face the demand, a conspicuous livestock trade has been developed from Mauritania, Algeria, and Mali to the refugee camps, and between the camps and the ‘liberated territories’ (mainly their northern regions while far less livestock exchange has been reported from the southern areas, where herders maintain a more traditional and less commercial animal husbandry system) [15]. These animals are sold in market areas of refugee camps, where they are slaughtered or incorporated into pre-existing flocks and herds. This substantial animal movement through trade with Mauritania and Mali could constitute a main route for virus spread. Recently, an outbreak of peste des petits ruminants has been reported in the wilayas territories and the origin likely ascribed to animal movements from neighbouring countries [33]. The last RVF outbreak was reported in Mauritania and Mali in 2003 [34,35], and more recently a RVF outbreak has erupted in the northern region of Mauritania, causing losses among people and cattle [36]. Therefore, the virus could have been introduced into the wilayas by infected animal trade, leading to low-level circulation after the rainfall event in 2006. During the interviews, Sahrawi reported the presence of the vectors in the territory (mainly in Smara and during the summer) and the persistence of flooding for months. These events may have constituted the necessary conditions for virus transmission, even though the arid ecosystem present in that region is not favourable for RVFV maintenance, in contrast to the Tifariti and Mehaires ecosystems. Low-level virus circulation may also occur between the refugee camps and the Tifariti region due to the movement of flocks between these areas during favourable seasons in relation to semi-nomadism of Sahrawi refugees and for marketing reasons.

## Conclusions

The presence of antibodies against the RVFV reported in this study could be a consequence of previous viral activity in the SADR, leading to concern that RVFV could be present in this territory. Investigating the health status of the animal population in a country or determining the level of disease risk is ideally carried out by the national line institutions within State policies framework. Unfortunately, the absence of functioning animal health institutions and specific policies for disease surveillance and control in some developing countries, such as the SADR, has been a major constraint for an extensive investigation of RVF. This highlights the need to allocate resources towards the establishment of RVF surveillance in SADR territories and a control programme capable of responding to disease occurrence. There is the need for further studies in order to assess the mechanism of possible RVF introduction into the SADR. The investigation revealed a scattered distribution of RVF seroprevalence, with the highest number of positive animals found in the Tifariti region. A monitoring system using resident sentinel herds in this region is therefore recommended.

## Abbreviations

ARRIVE: Animal Research Reporting of *In Vivo* Experiments; DEFF: Design Effect; ELISA: Enzyme-Linked Immunosorbent Assay; IgG: Immunoglobulin G; OD: Optical Density; PI: Percentage of Inhibition; ROH: Rate Of Homogeneity; RVF: Rift Valley Fever; RVFV: Rift Valley Fever Virus; SADR: Sahrawi Arab Democratic Republic; WS: Western Sahara.

## Competing interests

The authors declare that they have no competing interests.

## Authors’ contributions

ADN and MVT conceived and designed the study. ADN, DR, SMLM, SML and SJH implemented the field survey and collected the samples. ADN, DR and ADG performed the laboratory testing. GS participated in the coordination of the diagnostic testing. ADN and MVT analysed the data and drafted the manuscript. All authors read and approved the final manuscripts.
